# Serotonin effects on human iPSC-derived neural cell functions: from mitochondria to depression

**DOI:** 10.1038/s41380-024-02538-0

**Published:** 2024-03-26

**Authors:** Iseline Cardon, Sonja Grobecker, Frederike Jenne, Tatjana Jahner, Rainer Rupprecht, Vladimir M. Milenkovic, Christian H. Wetzel

**Affiliations:** Department of Psychiatry and Psychotherapy, University of Regensburg, 93053 Regensburg, Germany

**Keywords:** Neuroscience, Stem cells, Cell biology

## Abstract

Depression’s link to serotonin dysregulation is well-known. The monoamine theory posits that depression results from impaired serotonin activity, leading to the development of antidepressants targeting serotonin levels. However, their limited efficacy suggests a more complex cause. Recent studies highlight mitochondria as key players in depression’s pathophysiology. Mounting evidence indicates that mitochondrial dysfunction significantly correlates with major depressive disorder (MDD), underscoring its pivotal role in depression. Exploring the serotonin-mitochondrial connection, our study investigated the effects of chronic serotonin treatment on induced-pluripotent stem cell-derived astrocytes and neurons from healthy controls and two case study patients. One was a patient with antidepressant non-responding MDD (“Non-R”) and another had a non-genetic mitochondrial disorder (“Mito”). The results revealed that serotonin altered the expression of genes related to mitochondrial function and dynamics in neurons and had an equalizing effect on calcium homeostasis in astrocytes, while ATP levels seemed increased. Serotonin significantly decreased cytosolic and mitochondrial calcium in neurons. Electrophysiological measurements evidenced that serotonin depolarized the resting membrane potential, increased both sodium and potassium current density and ultimately improved the overall excitability of neurons. Specifically, neurons from the Non-R patient appeared responsive to serotonin in vitro, which seemed to improve neurotransmission. While it is unclear how this translates to the systemic level and AD resistance mechanisms are not fully elucidated, our observations show that despite his treatment resistance, this patient’s cortical neurons are responsive to serotonergic signals. In the Mito patient, evidence suggested that serotonin, by increasing excitability, exacerbated an existing hyperexcitability highlighting the importance of considering mitochondrial disorders in patients with MDD, and avoiding serotonin-increasing medication. Taken together, our findings suggested that serotonin positively affects calcium homeostasis in astrocytes and increases neuronal excitability. The latter effect must be considered carefully, as it could have beneficial or detrimental implications based on individual pathologies.

## Introduction

Depression is a severe mental disorder greatly affecting the patients’ quality of life. The earliest theory on the origins of depression relates to a monoamine deficiency in the brain and suggested a particularly significant role of serotonin in the etiology of the disease [1].

Serotonin deficiency in depression has been investigated for decades and has recently sparked renewed debates [2] inciting Jauhar et al. to review the most reliable abnormalities in serotonin in depressed patients [3]. The evidence includes findings from tryptophan depletion studies, indicating that reduced brain serotonin levels can trigger clinical relapse [3]. Additionally, impaired serotonin-mediated endocrine responses and lowered serotonin transporter binding in the raphe nucleus reinforce the serotonin-related pathology in depression [3].

In line with the proposed involvement of serotonin in depression, first-line antidepressants (ADs), selective serotonin reuptake inhibitors (SSRIs), are proposed to alleviate symptoms by increasing synaptic serotonin levels through reuptake inhibition. However, remission rate following primary AD treatment ranges between 30 and 45% [4]. Interestingly, an emerging strategy to treat resistant depression involves serotonergic psychedelics [5], which advocates for an involvement of serotonergic systems in the etiology of depression. Yet, doubts persist regarding whether the effect of ADs is genuinely mediated by increased synaptic serotonin [6], and the mechanisms underlying the antidepressant effect of psychedelics remain unclear. Therefore, investigating the effect of serotonin on brain cells is crucial.

Since the monoamine theory of depression, new theories emerged, implicating various biological factors including stress hormones, inflammatory cytokines [7], glucocorticoid neurotoxicity [8], decreased neurotrophic factors and neurogenesis [9], and altered GABAergic and glutamatergic neurotransmission [10].

Mitochondria play a central role in proposed biological processes, with growing evidence linking mitochondrial dysfunction, bioenergetic impairments, and major depressive disorder (MDD).

Neuroimaging studies in depressed patients found decreased ATP levels [11], which were reverted upon successful AD therapy [12]. Impaired mitochondrial function in cells from patients with MDD have been extensively documented, including decreased ATP production and respiratory enzymes activity in muscle cells [13], reduced mitochondrial respiration in platelets and immune cells [14, 15], and metabolic and energy production deficiencies in fibroblasts [16]. Moreover, decreased respiration in fibroblasts and neural progenitors cells (NPCs) [17, 18], and MDD-associated electrophysiological changes in neurons have been reported [18]. Post-mortem studies suggest that impairments observed peripherally are also present, potentially worse in the brain [19, 20]. For instance, a severely depressed patient exhibited significantly more mtDNA deletions in the brain compared to skeletal muscles [21], implying a potential contribution of mtDNA deletions in the brain to the pathophysiology of depression.

Growing evidence associate mitochondrial diseases (MDs) with psychiatric conditions, especially MDD. MD patients have a higher risk to develop a major mental illness. For instance, a study recorded that 63% of MD patients met the criteria for psychiatric illness, with 58% diagnosed with MDD [22]. Another reported a 70% lifetime prevalence of psychiatric illness in MD patients, with 54% having MDD [23]. Interestingly, psychiatric symptoms often precede the diagnosis of a MD by an average of 7.5 years and 14% of patients initially presenting with depressive symptoms are eventually diagnosed with MD [24]. Collectively, these findings suggest a central role of mitochondrial dysfunction in the pathophysiology of MDD.

Evidence is mounting that serotonin improves mitochondrial functions. Receptors 5-HT_3_ and 5-HT_4_, can localize on mitochondria and increase respiration and mitochondrial membrane potential (MMP) when activated by serotonin [25, 26]. They also impact mitochondrial Ca^2+^, reactive oxygen species and intracellular ATP [26]. Furthermore, serotonin signaling promotes mitochondrial biogenesis as seen in rat kidney cells with a 5-HT_2A_ agonist (DOI) [27, 28]. One study noted an upregulation of the mitochondrial biogenesis mediator PGC-1α [28]. The same effect was observed in mouse cells with a 5-HT_2B_-mediated mitochondrial biogenesis [29].

The investigation into the influence of serotonin on neuronal mitochondrial functions is relatively limited. Serotonin, the SSRI fluoxetine, and a 5-HT_1A_ agonist promote mitochondrial transport in hippocampal rat neurons [30]. In a Parkinson’s disease model, a 5-HT_1F_ agonist increased mitochondrial biogenesis and attenuated neuronal loss [31]. Additionally, in rat cortical neurons, serotonin and DOI increased mitochondrial biogenesis, respiration, ATP levels, antioxidant defenses, and these effects were mediated by the SIRT1- PGC-1α axis [32].

However, while the role of serotonin on mitochondria has been investigated in many animal models, research in human models, particularly in brain cells, remains relatively unexplored, necessitating further studies to bridge this gap in our understanding.

Considering the documented serotonin abnormalities in MDD and the beneficial effects of serotonin on mitochondrial functions, we posit a hypothesis proposing a potential link between mitochondrial impairments and serotonin disturbances in MDD. The exploration of this association in human brain cells holds significant promise for unraveling the intricate interplay between serotonin and mitochondrial health and might offer valuable insights into how they contribute to the development and progression of MDD.

We previously investigated cellular and mitochondrial functions in a cohort of MDD patients and non-depressed controls in fibroblasts, NPCs and neurons and demonstrated clear mitochondrial impairments and electrophysiological alterations [17, 18]. Next, building on these cohort studies, we performed an in-depth investigation of cellular and mitochondrial function in case study patients to extend our knowledge on the biological mechanisms underlying the development of MDD [33].

In this study, induced-pluripotent stem cell (iPSC)-derived astrocytes and neurons of the two case study patients and their matched controls were treated with serotonin for 6 days. The first patient, severely depressed and non-responsive to ADs, provided a unique case to examine mitochondrial and cellular parameters during active depression. This investigation also aimed to assess serotonin’s impact in cases resistant to standard treatment, offering potential insights into its role in depression. The second patient, with a mitochondriopathy but no depression symptoms, provided an opportunity to directly observe the relationship between serotonin and mitochondrial (dys)function in brain cells, isolated from the complexities of depressive pathology.

We differentiated astrocytes and neurons from these patients and their controls and analyzed gene expression, astrocytic respiration, cytosolic and mitochondrial Ca^2+^, mitochondrial membrane potential, Ca^2+^ transients, and electrophysiological parameters in neurons.

## Methods and materials

### Generation of control and patient iPSCs from fibroblasts

Human fibroblasts were obtained from patients and healthy age- and sex-matched controls and cultivated as described in ref. [17]. We refer to the non-responder patient as “Non-R” and to the mitochondriopathy patient as “Mito”. Their corresponding controls are “Ctl17” and “Ctl18”, respectively.

Fibroblasts were reprogrammed to iPSCs using the episomal protocol described in ref. [34]. More information can be found in the supplement.

### iPSC differentiation to NPCs and neuronal differentiation

iPSCs were differentiated to neural progenitor cells (NPCs) according to a monolayer culture protocol described in ref. [35].

NPCs from passage 5–12 were differentiated into cortical-like neurons for 21 days as described in ref. [33]. More information can be found in the supplement.

### Astrocytes differentiation

Astrocytes were differentiated from NPCs following a method adapted from ref. [36] and as described in ref. [33]. Astrocytes were used from day 30 to day 60 of differentiation. More information can be found in the supplement.

### Serotonin and serotonin receptor 5-HT_2A_ antagonist treatments

Serotonin hydrochloride (Sigma) was resuspended in water to a stock concentration of 100 mM and sterile-filtered. M100907 (volinanserin) (Sigma-Merck), a specific 5-HT_2A_ receptor antagonist, was resuspended in DMSO to a stock concentration of 10 mM.

Cells were treated with either 100 µM serotonin alone, or with a combination of 10 µM M100907 and 100 µM serotonin, or with 1:1000 DMSO and 100 µM serotonin for 6 days. Medium was refreshed after 3 days.

### RNA isolation, reverse transcription, and quantitative real-time RT-PCR

Quantitative RT-PCR experiments were performed as described in ref. [37] and in the supplement. A list of genes measured, corresponding protein names and primers sequences are provided in the supplement (Supplementation Table S1).

### Analysis of mitochondrial respiration

Mitochondrial respiration was analyzed using Seahorse XFp Flux analyzer with a Seahorse XFp Mito Stress Test Kit as described in ref. [33] and in the supplement.

### Immunofluorescence

Immunofluorescence staining was performed as described in ref. [18]. Information about antibodies is in the supplement.

### Luminescent assay for ATP content

We used CellTiter-Glo® Cell Viability Kit (Promega) to measure ATP content according to manufacturer’s instructions, as described in ref. [33] and in the supplement.

### Mitochondrial membrane potential (MMP), cytosolic and mitochondrial Ca^2+^

Live-cell imaging experiments were performed as described in ref. [33] and in the supplement. Cytosolic and mitochondrial Ca^2+^ were measured using Fura-2/AM and Rhod-2/AM, respectively. MMP was measured with JC-1. Additionally, cell size was measured in Fura-2/AM-loaded cells.

In neurons, after basal cytosolic Ca^2+^ measurements, spontaneous Ca^2+^ fluctuations were recorded over 20-min periods with 2 Hz frequency. Peaks were analyzed with the software IGOR Pro 9 (WaveMetrics).

### Electrophysiology

Whole-cell patch-clamp recordings were performed during the 4th week of neuronal differentiation as described in ref. [33] and in the supplement.

### Statistical analysis

Statistical analysis was conducted with Graph Pad Prism 9.5.1 (GraphPad Software). For all experiments, excluding patch-clamp recordings, means of three technical replicates and three biological replicates were averaged. A technical replicate refers to the repetition of the same experimental procedure multiple times on the same sample. Biological replicates refer to independent samples. Statistical outliers were detected and eliminated using ROUT-Method. Seahorse measurements were conducted pairwise, and a mixed-effect analysis one-way ANOVA was used. Imaging, ATP assay and patch-clamp experiments were analyzed using a one-way ANOVA without matching. qPCR experiments data were analyzed using unpaired t-test with Welch’s correction. Results are presented as mean ± SEM. *p*-value limit for statistical significance was set to ≤0.05.

## Results

### Serotonin altered mRNA expression in neurons and astrocytes

**Fig. 1:**
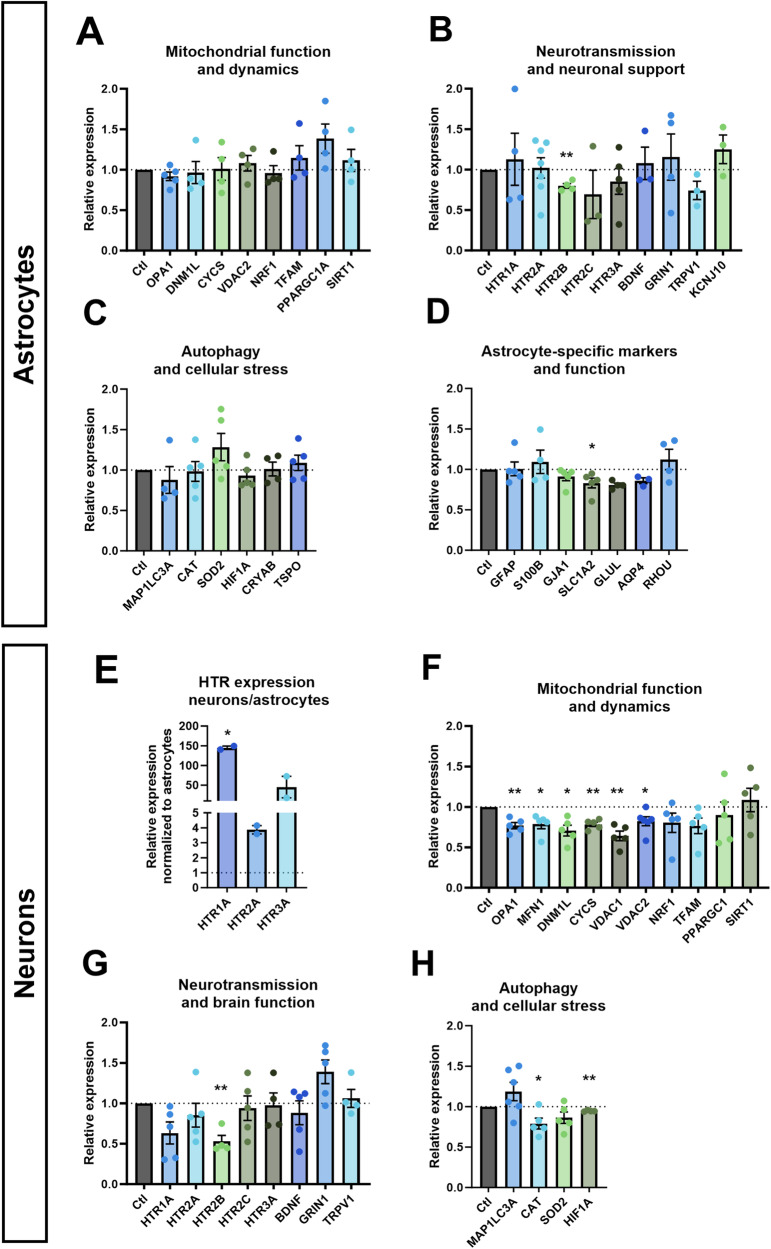
mRNA expression in astrocytes and neurons. mRNA expression of genes involved in **A** mitochondrial function and dynamics, **B** neurotransmission and neuronal support, **C** autophagy and cellular stress and **D** astrocyte-specific markers and function in serotonin-treated healthy astrocytes relative to untreated astrocytes. **E** mRNA expression of serotonin receptors 5-HT_1A_, 5-HT_1A_ and 5-HT_3A_ in untreated neurons relative to untreated astrocytes. mRNA expression of genes involved in **F** mitochondrial function and dynamics, **G** neurotransmission and brain function, and **H** autophagy and cellular stress in serotonin-treated healthy neurons relative to untreated neurons. All cells were derived from Ctl17. Ctl: untreated control. All data were analyzed using unpaired t-tests with Welch’s correction and presented as mean ± SEM. Significant differences were indicated with *p < 0.05, **p < 0.005.

We measured the effect of serotonin on the expression of key genes involved in mitochondrial function and dynamics, neurotransmission and neuronal support, autophagy and cellular stress, and astrocyte-specific markers and functions (Fig. 1A–D) in healthy astrocytes after a 6-days serotonin treatment. Serotonin induced a downregulation of receptor 5-HT_2B_ by 20% (Fig. 1B). SLC1A2, encoding the glutamate transporter EAAT2, was downregulated by 17% (Fig. 1D).

We compared the expression of HTR1A, 2A and 3A in neurons and astrocytes. In neurons, the expression of HTR1A was 145-fold higher, mostly due to low expression in astrocytes with a Ct of 27 compared to 16 in neurons. While HTR2A showed a more modest 4-fold increase in neurons, the expression was robust in both astrocytes and neurons with Ct values of 19 and 14, respectively. HTR3A exhibited a 45-fold increase relative to astrocytes, due to low expression in astrocytes with a Ct of 24, compared to a Ct of 16 in neurons (Fig. 1E).

Then, we measured the expression of genes involved in mitochondrial function and dynamics, neurotransmission and brain function, and autophagy and cellular stress (Fig. 1F–H). Several genes involved in mitochondrial function and dynamics were downregulated. The expression of OPA1 and MFN1 involved in mitochondrial fusion dropped by 23% and 21%, respectively. DNM1L, coding for the mitochondrial fission protein DRP1, was downregulated by 21%. CYCS, coding for cytochrome C in the electron transport chain (ETC) was downregulated by 22%. VDAC1 and VDAC2, involved in metabolite transport across the mitochondrial outer membrane, were downregulated by 36% and 18%, respectively (Fig. 1F). Additionally, like in astrocytes, the expression of the gene encoding serotonin receptor 5-HT_2B_ decreased by 47% (Fig. 1G). Regarding cellular stress response, the CAT gene encoding catalase was downregulated by 21% and the HIF-1α by 10% (Fig. 1H).

### Astrocytic respiration and ATP levels were mildly altered by serotonin

**Fig. 2:**
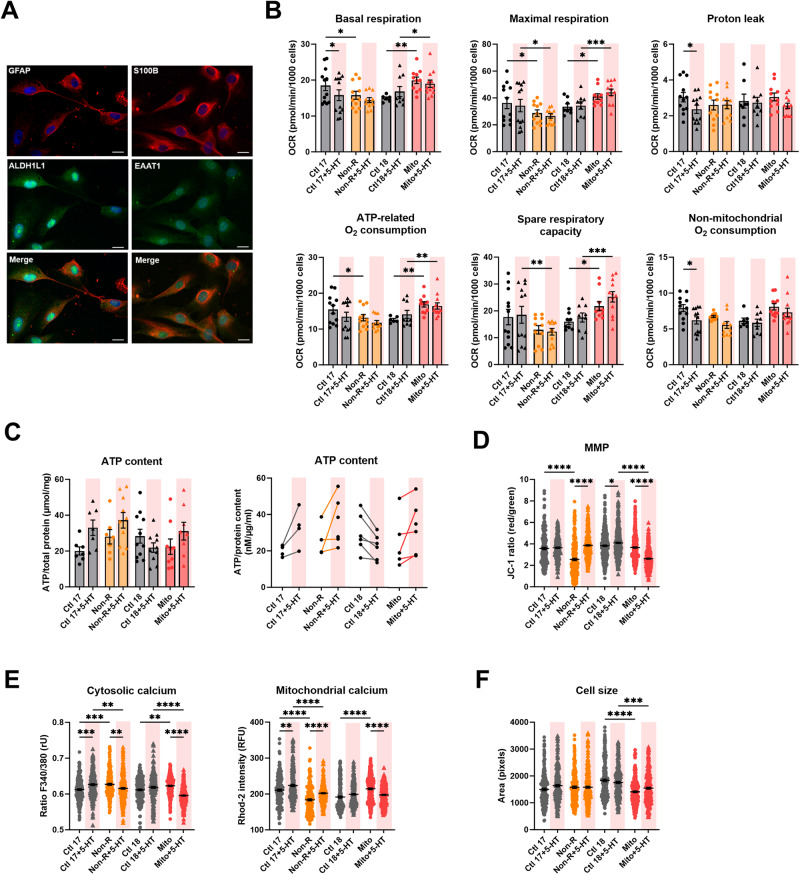
Effect of serotonin on the mitochondrial bioenergetics in astrocytes. **A** Astrocytes markers. Immunofluorescence stainings show that cells express the typical mature astrocytes markers GFAP, ALDH1L1, S100β, and EAAT1. Scale bar indicates 20 µm. **B** Mitochondrial respiration. The oxygen consumption rate (OCR) was measured in untreated and serotonin-treated astrocytes following the Agilent XF Mito Stress Test protocol consisting of sequential injections of oligomycin (Oligo), carbonyl cyanide-4-(trifluoromethoxy)-phenylhydrazone (FCCP) and rotenone/antimycin A (Rot/AntA) to reveal different respiratory parameters. OCR values were normalized to 1000 cells by counting DAPI-stained nuclei. Experiments were conducted pairwise allowing direct comparison. Bar plots show normalized mean OCR values ± SEM. **C** ATP content was measured in untreated and serotonin-treated astrocytes using a luminescent assay and normalized to protein amount. Left: bar plot shows nM ATP per µg/mL proteins ± SEM. Right: before-after graph shows nM ATP per µg/mL proteins in individual paired replicates, i.e. astrocytes from the same passage, harvested and measured at the same time. **D** Mitochondrial membrane potential (MMP) was measured with the JC-1 dye and is indicated by the fluorescence ratio between JC-1 aggregates (fluorescing in red) over JC-1 monomers (fluorescing in green). Dot plot shows mean red/green ratios ±SEM. **E** Cytosolic calcium was measured as the Fura-2 fluorescence ratio F340/380 and is represented as mean ratio ±SEM (left). Mitochondrial calcium levels were measured using Rhod-2/AM and are presented as mean fluorescence intensity, in relative fluorescent unit ±SEM (right). **F** Cell size was analyzed by assessing area (pixels) of Fura-2/AM-loaded cells. Dot plot shows the number of pixels ±SEM. Ctl17 and Ctl18: healthy controls; Non-R: non-responder patient; Mito: mitochondriopathy patient; 5-HT: serotonin. Respiration data were analyzed a mixed-effect analysis one-way ANOVA. ATP content, MMP and calcium imaging data were analyzed with a one-way ANOVA without matching or pairing. Significant differences were indicated with *p < 0.05, **p < 0.005, ***p < 0.0005) and ****p < 0.0001.

We investigated the effect of a chronic serotonin treatment in astrocytes. We generated mature and functional astrocytes as demonstrated by the expression of the specific astrocytic markers GFAP, S100β, ALDH1L1 and EAAT1 (Fig. 2A).

An effect of serotonin on mitochondrial respiration has been reported in several cell types [26, 32]. Therefore, we measured the oxygen consumption rates (OCR) in key respiratory states in our astrocytes cell lines. We used the Agilent’s Mito Stress Test, which involves the serial application of specific inhibitors of ETC complexes.

In our model, serotonin did not show a strong influence on mitochondrial respiration, apart from a significant effect on Ctl17 astrocytes: basal respiration, proton leak, and non-mitochondrial respiration decreased. Serotonin treatment did not alter respiration in the Ctl18/Mito pair and the Mito patient’s astrocytes presented increased OCR in all mitochondrial respiratory states but proton leak (Fig. 2B).

To complement the respiratory measurements, we analyzed cellular ATP levels. Although the serotonin treatment did not lead to statistically significant changes overall, a trend was observed when pairing individual data points. Specifically, there appeared to be an overall increase in ATP content in Ctl17, Non-R and Mito astrocytes. Contrastingly, ATP levels in the Ctl18 astrocytes seemed to decrease upon serotonin exposure (Fig. 2C).

### Serotonin influenced mitochondrial membrane potential and calcium homeostasis in astrocytes

The mitochondrial membrane potential (MMP) is an indirect measure of metabolic activity and capacity, reflecting the accumulation of protons in the intermembrane space. We assessed the MMP using the potential-dependent dye JC-1. Serotonin treatment increased the MMP of Non-R patient’s astrocytes, which effectively compensated the difference between Ctl17 and Non-R. In contrast, while serotonin increased the MMP in Ctl18, it markedly decreased it in Mito astrocytes resulting in a significantly lower MMP in Mito patient’s astrocytes (Fig. 2D).

Cellular Ca^2+^ homeostasis is crucial to mitochondrial function and plays an essential role in signaling, enzyme function, and metabolism. We investigated the effect of serotonin on astrocytic cytosolic and mitochondrial Ca^2+^ using Fura-2/AM and Rhod-2/AM, respectively. In Ctl17, serotonin increased cytosolic Ca^2+^ levels, whereas it decreased them in the Non-R patient. This effect reversed the difference between these two cell lines in untreated conditions. Similarly, serotonin treatment reversed the difference between Mito patient and Ctl18 astrocytes by decreasing cytosolic Ca^2+^ levels in the Mito patient’s astrocytes (Fig. 2E).

Furthermore, serotonin markedly increased mitochondrial Ca^2+^ levels in Ctl17 and Non-R astrocytes but decreased them in Mito astrocytes. While this increase was insufficient to normalize the significantly lower mitochondrial Ca^2+^ in Non-R, it did equalize the difference in mitochondrial Ca^2+^ levels between Ctl18 and Mito (Fig. 2E).

Serotonin did not alter cell size, measured in Fura-2/AM-loaded astrocytes. Mito patient’s astrocytes remained smaller than Ctl18 astrocytes (Fig. 2F).

In summary, serotonin significantly influenced Mito patient’s astrocytes by decreasing the MMP, cytosolic and mitochondrial Ca^2+^. In Non-R patient’s astrocytes, there was a consistent increase in MMP and mitochondrial Ca^2+^ levels after serotonin treatment. In Ctl17, serotonin increased both cytosolic and mitochondrial Ca^2+^, while it decreased the MMP in Ctl18.

### Serotonin decreased Ca^2+^ and altered the MMP in neurons

Research suggest that serotonin is dysregulated in the brain of depressed patients [3]. It is also suggested to have a prenervous, trophic role on neurons, and to improve mitochondrial biogenesis and function [32]. To investigate this in our model, we generated mature cortical-like neurons using a method adapted from ref. [35]. The neurons expressed MAP2, β-III-tubulin, NeuN, PSD95 and VGLUT1, confirming their maturity and their identity [38–41].

**Fig. 3:**
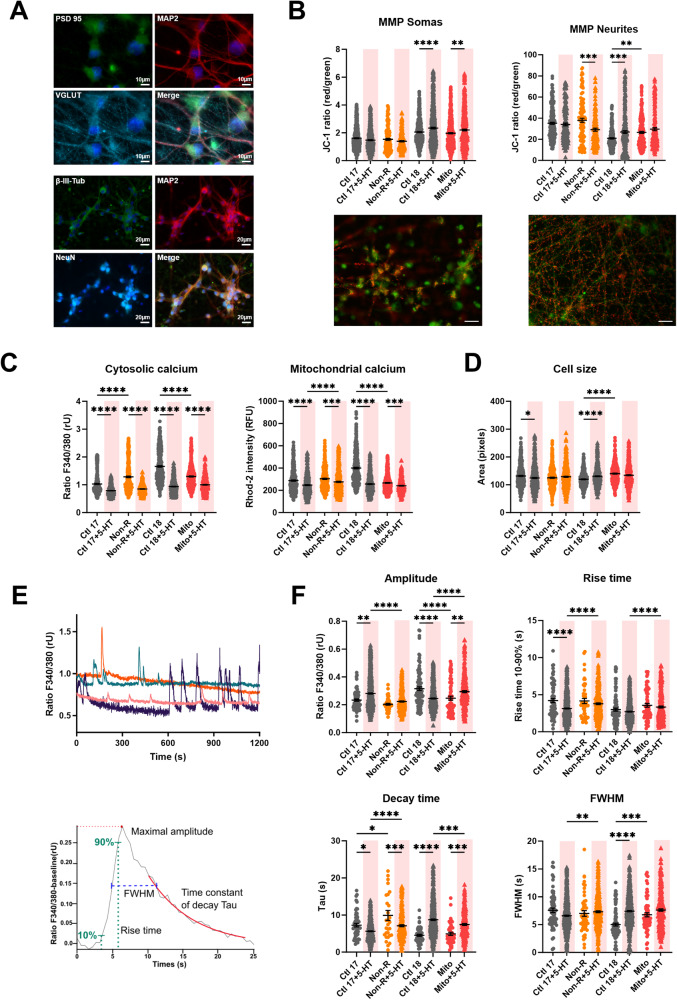
Effect of serotonin on mitochondrial membrane potential (MMP), calcium homeostasis and dynamics in iPS-Neurons. **A** Neuronal markers. Immunofluorescence stainings on untreated control neurons revealed that the induced neurons express typical neuronal cytoskeleton proteins MAP2 and βIII-Tubulin and neuronal nuclear marker NeuN. PSD95 and VGLUT1 expression indicated the presence of mature synaptic terminals. VGLUT1 expression suggested that most of the induced neurons are cortical-like glutamatergic neurons. Scale bars indicate 20 µm or 10 µm. **B** MMP was measured with the JC-1 dye and is indicated by the fluorescence ratio between JC-1 aggregates (fluorescing in red) over JC-1 monomers (fluorescing in green). Mitochondria from somas and neurites appeared on different focal planes and were therefore imaged separately. Representative images show red and green JC-1 fluorescence in the relevant structure. Scale bar indicates 20 µm. Dot plot shows mean red/green ratios ±SEM. **C** Calcium homeostasis in untreated and serotonin-treated neurons. Cytosolic calcium was measured as the Fura-2 fluorescence ratio F340/380 and is represented as mean ratio ±SEM. Mitochondrial calcium levels were measured using Rhod-2/AM and are presented as mean fluorescence intensity, in relative fluorescent unit ±SEM. **D** Cell size was analyzed by assessing area (pixels) of Fura-2/AM-loaded cells. Dot plot shows the number of pixels ±SEM. **E** Spontaneous calcium transients were analyzed in Fura-2/AM-loaded neurons. Example traces show representative calcium transients in a neuron (above) and a baseline subtracted calcium peak, illustrating maximal amplitude, rise time between 10 and 90% of maximal amplitude, the exponential fit used to calculate the time constant of decay Tau, and the full-width at half maximum (FWHM) (below). **F** Calcium transient dynamics in untreated and serotonin-treated neurons. Graphs show the maximum amplitude of the calcium transients (ratio 340 nm/380 nm ±SEM), the rise time, the time constant of decay Tau (s ± SEM) and the FWHM (s ± SEM). Ctl17 and Ctl18: healthy controls; Non-R: non-responder patient; Mito: mitochondriopathy patient; 5-HT: serotonin. All data were analyzed with a one-way ANOVA without matching or pairing. Significant differences were indicated with *p < 0.05, **p < 0.005, ***p < 0.0005 and ****p < 0.0001.

As an indicator of mitochondrial function, we assessed the MMP in both somas and neurites using JC-1. In somatic mitochondria, serotonin increased MMP in both Ctl18 and Mito neurons. However, in neurites, serotonin treatment decreased the MMP in Non-R neurons, but increased it in Ctl18 neurons. As a result, the MMP was no longer higher in Mito neurons’ neurites compared to Ctl18 (Fig. 3B).

Ca^2+^ is an important second messenger involved in serotonin’s signaling through G_q_ protein-coupled receptors [42]. Moreover, Ca^2+^ homeostasis is crucial to mediate neurotransmission, and mitochondria play a pivotal role in balancing intracellular Ca^2+^ levels. Therefore, we investigated the effect of serotonin on Ca^2+^ homeostasis in neurons.

Serotonin consistently decreased both cytosolic and mitochondrial Ca^2+^ levels across all cell lines. For cytosolic Ca^2+^, this led to a normalization of differences between control and patient cells. For mitochondrial Ca^2+^, serotonin treatment resulted in higher levels in Non-R neurons compared to the matched Ctl17, but balanced the difference between Ctl18 and the Mito patient (Fig. 3C).

Serotonin decreased cell size in Ctl17 neurons but increased it in Ctl18 neurons, thereby eliminating the difference with Mito neurons (Fig. 3D).

Overall, serotonin markedly decreased Ca^2+^ levels in both the cytosol and mitochondria, while its impact on MMP varied. Notably, serotonin treatment often compensated for the differences between control and patient neurons, indicating an equalizing effect.

### Serotonin altered the kinetics of Ca^2+^ transients

Besides measuring basal cytosolic Ca^2+^ levels, we recorded spontaneous Ca^2+^ transients over 20-minutes periods. We analyzed singles peaks to extract their amplitude, rise time, decay time and full width at half maximum (FWHM) (Fig. 3E).

The amplitude was increased by serotonin in Ctl17 and Mito neurons but decreased in Ctl18 neurons. This led to lower Ca^2+^ amplitudes in Non-R neurons and higher amplitudes in Mito neurons (Fig. 3F).

The rise time was markedly shortened by serotonin in Ctl17 neurons. Consequently, Non-R neurons exhibited a significantly longer rise time than Ctl17. The relative difference between Ctl18 and Mito remained similar before and after treatment, with longer rise time in Mito (Fig. 3F).

Serotonin prolonged the decay time in all cell lines but Ctl18, resulting in a lower rise time in Mito neurons, relative to Ctl18 (Fig. 3F).

The FWHM, measuring the duration of Ca^2+^ peaks, increased in Ctl18 upon serotonin treatment, normalizing the difference between Ctl18 and Mito (Fig. 3F).

In summary, serotonin treatment had varied effects on the kinetics of Ca^2+^ peaks. In Ctl17 neurons, serotonin made Ca^2+^ peaks higher, shortened rise time but prolonged decay time. In Non-R neurons, serotonin shortened decay time. In Ctl18 neurons, serotonin decreased amplitude but increased decay time and FWHM, resulting in lower and wider peaks. In Mito neurons, serotonin increased both the amplitude and the decay time of Ca^2+^ transients. Collectively, these findings demonstrate that serotonin had marked effects on spontaneous Ca^2+^ transients kinetics, although the effects varied among cell lines.

### Serotonin depolarized RMP, decreased capacitance and increased current densities in neurons

Electrical activity is a hallmark of neuronal function. To investigate how serotonin influenced the biophysical properties of neurons, we performed whole-cell patch-clamp recordings. Moreover, 5HT_2A_ signaling has been proposed to mediate serotonin’s effect on neuronal mitochondria [32]. Therefore, we additionally treated neurons with the specific 5HT_2A_ antagonist M100907 (volinanserin) to test whether 5HT_2A_ also mediated the effects of serotonin on electrophysiology.

**Fig. 4:**
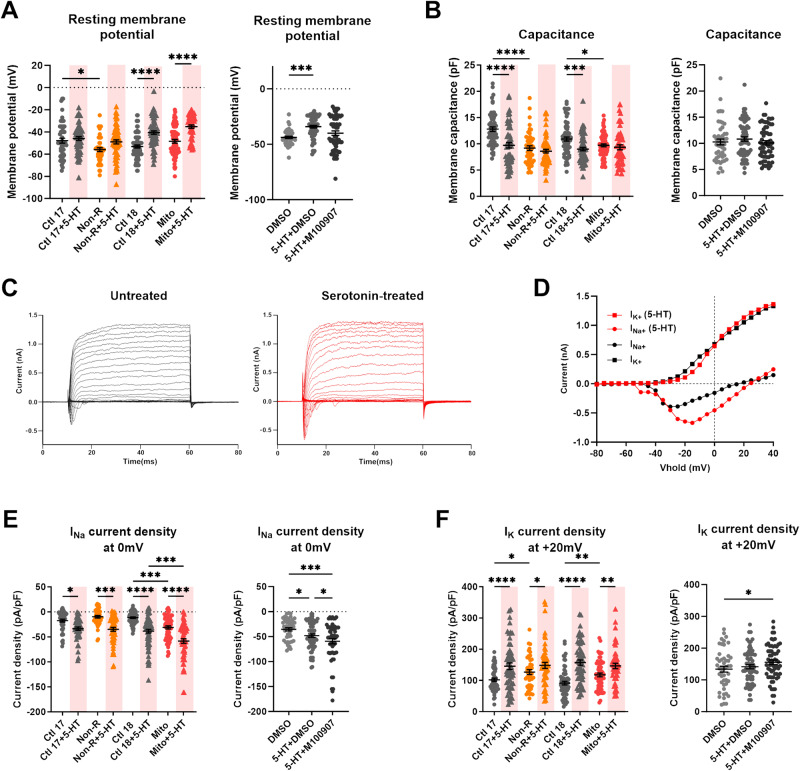
Effect of serotonin and 5-HT_2A_ antagonist M100907 on electrophysiological properties of iPS-Neurons. **A** Resting membrane potential (RMP) in untreated and serotonin-treated neurons (left) and in DMSO-, serotonin+DMSO- and serotonin+M100907-treated neurons (right). Dot plots show mean RMP in mV ± SEM. **B** Membrane capacitance in untreated and serotonin-treated neurons (left) and in DMSO-, serotonin+DMSO- and serotonin+M100907-treated neurons (right). Dot plots show mean capacitance in pF ± SEM. **C**, **D** Sodium (I_Na_) and potassium (I_K_) currents were recorded in voltage-clamp mode while holding the membrane potential at −80 mV (Vhold) and depolarizing in steps of 10 mV to provoke the opening of voltage-gated Na^+^ and K^+^ channels. Example traces show (**C**) the evoked Na^+^ and K^+^ current in untreated (black) and serotonin-treated (red) neurons, and (**D**) the resulting IV curves. **E**, **F** Sodium and potassium current densities at 0 mV in untreated and serotonin-treated neurons (left) and in DMSO-, serotonin+DMSO- and serotonin+M100907-treated neurons (right). Current measurements were normalized to the membrane capacitance to account for cell size variability (current density, pA/pF). **E** Dot plots show mean I_Na_ current density at 0 mV in pA/pF ± SEM and **F** mean I_K_ current density at +20 mV in pA/pF ± SEM. Ctl17 and Ctl18: healthy controls; Non-R: non-responder patient; Mito: mitochondriopathy patient; 5-HT: serotonin. All data were analyzed with a one-way ANOVA without matching or pairing. Significant differences were indicated with *p < 0.05, **p < 0.005, ***p < 0.0005 and ****p < 0.0001.

Serotonin induced a significant depolarization of the resting membrane potential (RMP) in Ctl18 and Mito neurons. In Ctl17 and Non-R neurons, the RMP was also depolarized. This change was sufficient to normalize the difference between Non-R and Ctl17 neurons’ RMP. Remarkably, the depolarizing effect was lost when combining serotonin with M100907, indicating that it was mediated by 5-HTR_2A_ signaling (Fig. 4A).

Serotonin decreased membrane capacitance in Ctl17 and Ctl18. This equalized capacitance between control and patient neurons after treatment. Notably, we previously reported consistently smaller cell size in MDD and case study patients [33]. M100907 had no effect on capacitance (Fig. 4B).

We also measured active electrophysiological parameters, including sodium (Na^+^) and potassium (K^+^) currents. By holding the membrane potential at −80 mV and depolarizing it in steps of 10 mV, we induced the opening of voltage-gated Na^+^ and K^+^ channels (Fig. 4C, D). We normalized the currents to the soma size, reflected by membrane capacitance, resulting in current densities (pA/pF).

Remarkably, serotonin treatment consistently increased Na^+^ and K^+^ current densities in all cell lines. These changes did not alter the control/patient differences in Na^+^ currents. Contrastingly, the higher K^+^ currents observed in untreated Non-R and Mito neurons were equalized to the level of their controls. Interestingly, co-treatment with serotonin and M100907 led to a further increase in both Na^+^ and K^+^ current densities, however the underlying mechanism is not clear (Fig. 4E, F).

### Serotonin altered the kinetics spontaneous postsynaptic currents and action potentials

**Fig. 5:**
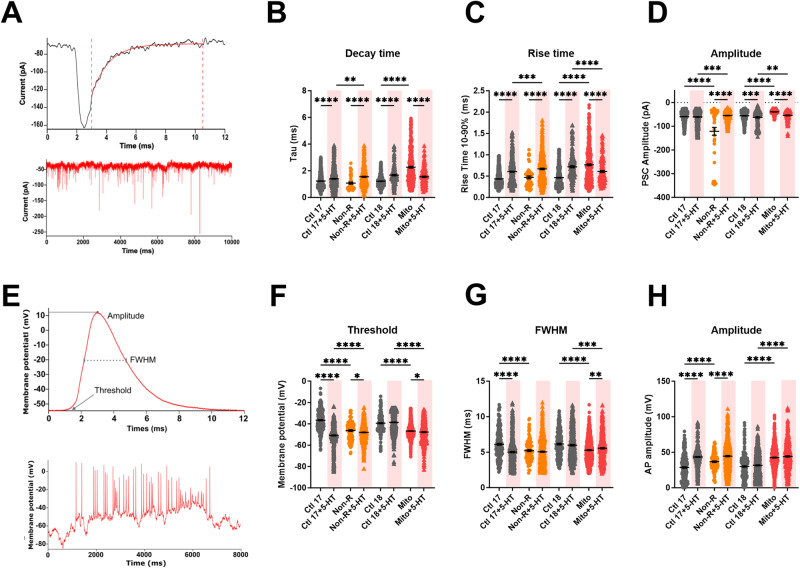
Effect of serotonin on postsynaptic currents and action potentials kinetics. **A** Spontaneous post-synaptic currents (PSCs) were recorded at a holding potential of −80 mV. Example traces show one single PSC (above) spontaneous PSCs (below). Graphs show **B** the time constant of decay Tau (ms ± SEM), **C** the rise time between 10% and 90% of the maximal amplitude, and **D** the maximum amplitude of the PSCs (pA ± SEM) in untreated and serotonin-treated neurons. **E** Spontaneous APs at −80 mV were analyzed individually. Example traces show a single AP trace illustrating threshold, amplitude and full width at half maximum (FWHM) (above) and spontaneous APs (below). Graphs show **F** mean threshold in mV ± SEM, **G** mean FWHM in ms ± SEM and **H** mean AP amplitude in mV ± SEM in untreated and serotonin-treated neurons. Ctl17 and Ctl18: healthy controls; Non-R: non-responder patient; Mito: mitochondriopathy patient; 5-HT: serotonin. All data were analyzed with a one-way ANOVA without matching or pairing. Significant differences were indicated with *p < 0.05, **p < 0.005, ***p < 0.0005 and ****p < 0.0001.

Next, we held the membrane potential at −80 mV and recorded the current fluctuations corresponding to postsynaptic currents (PSCs) (Fig. 5A). We measured the decay time, rise time and amplitude of PSCs.

Serotonin had a marked influence on the kinetics of PSCs. Specifically, serotonin prolonged both the rise time and the decay time of the PSCs in all individuals but the Mito patient, where the effect was reversed. This resulted in prolonged PSCs in the Non-R and the Mito patient compared to their respective controls (Fig. 5B, C). On the other hand, serotonin increased the amplitude of PSCs in both Ctl18 and Mito neurons, maintaining the difference between them. Contrastingly, the amplitude decreased in serotonin-treated Non-R patient’s neurons, which made them smaller than in Ctl17 neurons (Fig. 5D).

We also investigated the effect of serotonin on spontaneous action potentials (APs). Therefore, we injected current to adjust the membrane potential to approximately −80 mV. We measured the threshold, FWHM and amplitude of the APs (Fig. 5E).

Serotonin decreased the AP threshold in all individuals but Ctl18. As a result, the difference between Ctl17 and Non-R was reversed, while Mito retained a lower threshold than Ctl18 (Fig. 5F). Serotonin reduced the width of the APs in Ctl17 neurons, which normalized its difference with Non-R neuron’s APs. In contrast, serotonin increased FWHM in Mito patient’s neurons, although insufficiently to equalize it to Ctl18 levels (Fig. 5G). Finally, APs amplitude increased in Ctl17 and Non-R neurons after serotonin treatment, which compensated the difference between them. Contrastingly, serotonin did not affect Ctl18 and Mito neurons’ APs (Fig. 5H).

## Discussion

The aim of this study was to investigate the effect of a chronic serotonin treatment on astrocytes and neurons derived from healthy controls and patients, focusing on mitochondrial function and electrophysiology. The two case study patients were selected for our previous study exploring the ways in which mitochondria can influence cellular function and contribute to the development of depression [33].

The relative expression of serotonin receptors was significantly higher in neurons compared to astrocytes, especially HTR1A. Notably, while HTR1A and HTR3A expression were low in astrocytes, both neurons and astrocytes robustly expressed HTR2A. This suggest that the effects of serotonin in astrocytes were mainly mediated by 5-HT_2A_ signaling. All three serotonin receptors had a similar expression in neurons.

In astrocytes, serotonin downregulated SLC1A2 and HTR2B, encoding the glutamate transporter EAAT2 and the 5-HT_2B_ receptor, respectively. The downregulation of 5-HT_2B_ suggests it had an additional role in mediating serotonin effects in astrocytes and may have been downregulated as a compensatory mechanism. Notably, Chen et al. reported that stimulation of the 5-HT_2B_ receptor in astrocytes increases glutamate production [43]. It is therefore conceivable that the downregulation of both SLC1A2 and HTR2B in response to serotonin might be functionally interrelated, although further studies are needed to explore this potential connection.

Serotonin treatment led to significant alterations in neuronal gene expression. The downregulation of OPA1, MFN1 and DNM1L suggests reduced mitochondrial fusion and fission. While this could be indicative of a compromised mitochondrial network, it may also represent an adaptation to mitigate the impact of cellular stressors. The downregulation of cytochrome C, VDAC1 and VDAC2 mRNA also suggest an impact of serotonin on the metabolic state of neurons. Noteworthy, our results contrast with findings presented by Fanibunda et al. in rat primary neurons, where serotonin treatment exerted a positive trophic effect accompanied by an upregulation of PPARGC1A and SIRT1 expression [32]. HIF-1α downregulation could alter glucose metabolism in neurons. Notably, Shibata et al. have observed trait-dependent expression levels of HIF-1α mRNA in MDD patients’ white blood cells, with remissive patients showing a decreased HIF-1α expression [44]. It is possible that in the latter study, increased serotonin from SSRI medication provoked the HIF-1α downregulation, and that the same mechanism was responsible for the serotonin-mediated HIF-1α downregulation we observed. However, the precise mechanism and its implications requires further investigation.

A notable effect of serotonin on astrocyte respiration was observed only in Ctl17 where several respiratory parameters decreased. This aligns with findings in the rat brain [45]. Contrastingly, Fanibunda et al. found that serotonin increased respiration in rat primary neurons [32]. Assuming that respiration rates are comparable in neurons and astrocytes [46], the decreased respiration in whole brain and certain of our astrocyte, coupled with the reported increase in neurons, suggests a potential opposing effect of serotonin on mitochondrial respiration in astrocytes and neurons. Technical constraints prevented the direct measurement of neuronal OXPHOS in our study. Exploring this aspect in future research would be valuable. Additionally, it is important to consider potential metabolic differences between rodent and human brain cells. They could partly account for discrepancies between our observations and reports in the literature. To the best of our knowledge, this is the first study investigating the effect of serotonin on the mitochondrial and cellular function of human astrocytes.

Although serotonin decreased or did not alter astrocytic respiration in our human model, it seemed to increase ATP levels, suggesting a glycolysis elevation. Consistently, studies showed that serotonin increases glucose uptake by increasing surface expression of glucose transporters [47], and that it stimulates glycolysis by upregulating and activating the rate limiting enzyme phosphofructokinase [48]. Future studies investigating the rate of glycolysis and glucose uptake in serotonin-treated astrocytes would be insightful.

Serotonin altered cytosolic and mitochondrial Ca^2+^ levels in astrocytes in a way that may suggest an equalizing effect. Indeed, while both the Non-R and the Mito patient initially exhibited elevated cytosolic Ca^2+^ levels relative to their matched controls, serotonin treatments significantly reduced Ca^2+^ in these patients’ astrocytes. Further supporting a normalizing influence of serotonin on altered Ca^2+^ homeostasis, low mitochondrial Ca^2+^ levels in the Non-R patient increased with serotonin treatment, while elevated Ca^2+^ in the Mito patient decreased. Serotonin receptors from the 5-HT_2_ family are coupled to phospholipase C (PLC) signaling and lead to increased Ca^2+^ levels both by mobilization of internal stores and opening of Ca^2+^ channels. These mechanisms could play a role in the observed compensatory effect of serotonin.

Remarkably, in neurons, serotonin consistently decreased Ca^2+^ levels in all cell lines and slowed Ca^2+^ transients. Lower cytosolic Ca^2+^ levels could result from an upregulation of the plasma membrane Ca²^+^-ATPase or the sodium-calcium exchanger, leading to enhanced Ca^2+^ efflux. Decreased cytosolic Ca^2+^ and slower Ca^2+^ transients in neurons can influence neurotransmission and impact Ca^2+^-mediated short-term plasticity mechanisms. Decreasing Ca^2+^ may also be a mechanism to prevent excitotoxicity.

In addition to cytosolic Ca^2+^, serotonin consistently decreased mitochondrial Ca^2+^ levels. This could result from lower cytosolic Ca^2+^ levels to be taken up through the mitochondrial Ca^2+^ uniporter or be related to the decreased VDAC expression in serotonin-treated neurons. Indeed, VDAC is considered as the main entry point of Ca^2+^ into mitochondria [49]. Moreover, mitochondrial distribution, partly mediated by fusion and fission, influences mitochondrial Ca^2+^ levels [50]. Therefore, decreased mitochondrial dynamics, as suggested by mRNA expression, could play a role in the lower Ca^2+^ levels. A decrease in mitochondrial Ca^2+^ levels can significantly impair OXPHOS, and therefore ATP production in neurons.

Serotonin had a consistent depolarizing effect on resting membrane potential (RMP), especially marked in Ctl18 and Mito patient’s neurons. Notably, the inhibition of that effect by M100907, a specific 5HT_2A_ receptor antagonist, suggested it was mediated by 5HT_2A_ signaling. Neurons with depolarized RMP would require smaller stimuli to reach the threshold for action potential (AP) generation. Therefore, serotonin appears to render cortical neurons more excitable. Aligning with this, slice recordings from prefrontal cortical neurons indicate depolarizing effects following 5-HT_2A_ receptor activation [51].

5HT_2A_ receptor stimulates PLC, which hydrolyses phosphatidylinositol (4,5) bisphosphate (PIP_2_) to produce inositol triphosphate (IP_3_) and diacylglycerol (DAG). DAG and the IP_3_-mediated Ca^2+^ release activate protein kinase C (PKC) [42]. Two components of the G_αq_ signaling cascade are known to modulate ionic channels and could influence the RMP.

First, PKC regulates ionic channels through mechanisms including altered surface expression and probability of opening [52]. Fryckstedt et al. demonstrated that serotonin decreased the activity of the Na^+^/K^+^ ATPase in a PKC-dependent manner [53]. This pump is particularly significant for maintaining a hyperpolarized RMP. Thus, a decrease in its activity could contribute to a depolarized RMP.

Second, PIP_2_ in the plasma membrane appears to control channel gating. For instance, PIP_2_ activates the two-pore domain potassium (K_2P_) channels, which mediate K^+^ background currents that maintain a hyperpolarized RMP [52]. Therefore, a plausible mechanism underlying the depolarizing effect of serotonin involves the activation of PLC via the 5-HT_2A_ receptor, resulting in PIP_2_ hydrolysis, decreasing the activation of K_2P_ channels, and consequently depolarizing the RMP.

Serotonin had a remarkable effect on current densities in neurons: both Na^+^ and K^+^ current densities were significantly increased in all cell lines. These results suggest that serotonin-treated neurons undergo faster depolarization and repolarization during APs. This could have profound implications on synaptic transmission.

This effect was not mediated by the 5HT_2A_ receptor, as it was exacerbated byM100907. This may suggest that a constitutive activity of 5HT_2A_ receptor partly mitigated the serotonin-mediated increase in Na^+^ and K^+^ current density, and that the antagonist lifted that restriction. Constitutive activity of 5HT_2A_ receptors has been reported before and is interestingly believed to play a role in the etiology of depression and in the effect of anti-depressive therapies [54].

Serotonin also prolonged the rise time and decay time of postsynaptic currents (PSCs) in most cell lines. This could affect the integration of events at the soma and lead to an increased summation if the broader excitatory PSCs overlap. Overall, this suggests a stronger effect of the PSCs and therefore an increased excitability.

AP kinetics were also altered by serotonin. The lower threshold observed in most cell lines is consistent with increased Na^+^ current density and could also indicate a shift in the activation properties of voltage-gated Na^+^ channels. It suggests weaker stimuli can induce APs. Taken together with a depolarized RMP, serotonin seems to generally increase neurons’ excitability. Higher Na^+^ current density could also underlie the serotonin-induced increased AP amplitude in Ctl17 and Non-R neurons.

## Limitations

Interindividual differences could be exacerbated by the differences in the age of the patient/control pairs [33].

We recognize that our results reflect the observations of independent individuals and cannot be interpreted as generally applicable interpretations of MD and TRD.

Reprogramming fibroblasts may affect the expression of disease-associated epigenetic memories. However, we have already shown that functional mitochondrial phenotypes are transmitted (at least partially) to the iPS-derived lineages [18, 33].

While our cellular models have provided valuable insights into mitochondrial dysfunction in MDD pathophysiology, we recognize the inherent complexity of in vivo systems. Extrapolating findings from isolated cellular contexts to the intricacies of whole organisms involves inherent limitations.

## Conclusion

Serotonin seems to have an equalizing effect on Non-R patient’s astrocytes, which compensated for the difference between the patient and its matched control in cytosolic and mitochondrial Ca^2+^ levels and MMP. In neurons, serotonin depolarized the negative RMP, increased Na^+^ and K^+^ currents, prolonged PSCs, made APs higher and lowered their threshold. Taken together, these effects improve excitability and therefore facilitate neurotransmission. While it is unclear how this translates to the systemic level and AD resistance mechanisms are not fully elucidated, our observations show that despite his treatment resistance to ADs, this patient’s cortical neurons are responsive to serotonergic signals.

In the Mito patient, serotonin also seemed to have a normalizing effect on astrocytes by increasing ATP levels and decreasing Ca^2+^ levels and MMP. In neurons, serotonin increased Ca^2+^ entry by increasing the amplitude and prolonging the Ca^2+^ transients. Additionally, it further depolarized the RMP, increased Na^+^ and K^+^ currents, made PSCs faster and bigger, made APs wider and lowered their threshold. Together, these findings suggest a heightened excitability. We previously demonstrated that Mito patient’s neurons were hyperexcitable, which is a common feature in mitochondrial pathologies [33, 55, 56]. Notably, SSRIs can impair mitochondrial function [57] and cause clinical deterioration in patients with mitochondrial disorders [58, 59]. In a striking case study, a depressed patient’s symptoms worsened with SSRIs treatment, until she was diagnosed with the MELAS syndrome, notably characterized by hyperexcitability, and symptoms improved drastically upon SSRI treatment termination [58]. Based on our findings, the effect of serotonin on neuronal excitability could explain these observations. This highlights the importance of considering mitochondrial disorders in patients with MDD and avoiding serotonin-increasing medication.

Overall, serotonin increased ATP levels and compensated Ca^2+^ alterations in patients, suggesting improved cellular function. In neurons, serotonin altered the expression of genes related to mitochondrial functions, decreased Ca^2+^, depolarized the RMP in a 5-HT_2A_-dependent manner, increased Na^+^ and K^+^ current densities, prolonged PSCs and lowered the threshold for AP generation. Together, these effects indicate the serotonin increased neurons’ excitability.

## Supplementary information

Supplemental Material

## Data Availability

The datasets generated during and/or analyzed during the current study are available from the corresponding author on request.
