# Supplementary material for: Serotonin effects on human iPSC-derived neural cell functions: from mitochondria to depression
Source: Mol Psychiatry. 2024 Mar 26;29(9):2689–700. doi: 10.1038/s41380-024-02538-0 (PMC11420088; doi:10.1038/s41380-024-02538-0)
Supplement: Supplementary file 1 — Supplemental Material [file 41380_2024_2538_MOESM1_ESM.docx]

**Table S1.** Primer sets used for QRT-PCR

| Gene name | Protein name | Forward primer (5´-3´) | Reverse primer (5´-3´) |
| --- | --- | --- | --- |
| CAT | Catalase (CAT) | CTCCGGAACAACAGCCTTCT | ATAGAATGCCCGCACCTGAG |
| CRYAB | Alpha-crystallin B chain (CRYAB) | TGCTTCACATCCAGGTTGAC | CAGCTGGTTTGACACTGGAC |
| CYCS | Cytochrome c (CYCS) | CGTTGAAAAGGGAGGCAAGC | ATTGGCGGCTGTGTAAGAGT |
| DNM1L | Dynamin-1-like protein (DNM1L) | TATGCCAGCCAGTCCACAAA | CACAATCTCGCTGTTCCCGA |
| GFAP | Glial fibrillary acidic protein (GFAP) | CAGGAAGCTGCTAGAGGGC | CCCCGTCTTTGGTGCTTTTG |
| GJA1 | Gap junction alpha-1 protein (Connexin 43) (GJA1) | TGAGTGCCTGAACTTGCCTT | CTCCAGTCACCCATGTTGCC |
| GLUL | Glutamate-ammonia ligase (Glutamine synthetase) (GLUL) | GATGCAAGATGAAACGGGCC | TGCTGGAGTCAAGATTGCGG |
| GRIN1 | Glutamate receptor ionotropic, NMDA 1 (GRIN1) | CAGGCGGAGAGACAGAGAAG | CATGTCCCATCACTCAGCGT |
| HIF1A | Hypoxia-inducible factor 1-alpha (HIF1A) | TGGACTCTGATCATCTGACCA | CCACCTCTTTTGGCAAGCAT |
| HTR1A | Serotonin receptor 1A (HTR1A) | TCAATTGGCTGGGCTACTCC | TGGCGGCAGAACTTACACTT |
| HTR2A | Serotonin receptor 2A (HTR2A) | ACAAGGAAACCCAGCAGCAT | TCGTCATCATGGCAGTGTCC |
| HTR2B | Serotonin receptor 2B (HTR2B) | GCCAATTGCCCTCTTGACAA | GCGGTTGAAAAGAGAACGTCA |
| HTR2C | Serotonin receptor 2C (HTR2C) | CTAACACCCGCGAGCATCTA | AGACGAAATTAACAGGCTAGGT |
| HTR3A | Serotonin receptor 3A (HTR3A) | CACCAAGTTGTCCATCCCCA | ACTTCCCCACATCCACGAAAC |
| KCNJ10 | Potassium inwardly-rectifying channel, subfamily J, member 10 (KCNJ10) | CACCTCGGACCCAAGATGAC | TCGTATCCCTGGGCCCATTA |
| MAP1LC3A | Microtubule-associated proteins 1A/1B light chain 3A (MAP1LC3A) | CGCCGGATGATCTTGACCAA | GGTGATCATCGAGCGCTACA |
| MFN1 | Mitofusin-1 (MFN1) | AGGATGATTGTTAGCTCCACGA | CACAGGCGAGCAAAAGTGG |
| NRF1 | Nuclear respiratory factor 1 (NRF1) | GAATTGCCAACCACGGTCAC | CTCACCTCCCTGTAACGTGG |
| OPA1 | Optic atrophy 1 protein (OPA1) | TGGAAGATTTTGCTGAAGATGGT | GGTCTTCCGCCAGTTGAACG |
| PPARGC1A | Peroxisome proliferator-activated receptor gamma coactivator 1-alpha (PPARGC1A) | CAAGCAAAGGGAGAGGCAGA | GTCCCTCAGTTCTGTCCGTG |
| RHOU | Rho-related GTP-binding protein RhoU (RHOU) | CCCACCGAGTACATCCCTAC | CAAATTCATCCTGTCCGGCA |
| S100B | S100 calcium-binding protein B (S100B) | CTTGTGCTTGTCTCCCTCCC | CAGGAAGGGGTGAGACAAGG |
| SIRT1 | Sirtuin 1 (SIRT1) | CCAGCCATCTCTCTGTCACA | CAACCTGTTCCAGCGTGTCT |
| SLC1A2 | Solute carrier family 1 member 2 (SLC1A2) or Excitatory amino acid transporter 2 (EAAT2) | CCCCAAAAGAGTCACCCACA | TCCTCATTCTGACAGCCGTG |
| TRPV1 | Transient receptor potential cation channel subfamily V member 1 (TRPV1) | CCTTGAGGTGGCTGAAGTAC | CGATGAAGACCCTGTTTGTGG |
| TFAM | Mitochondrial transcription factor A (TFAM) | AGAAGAATTGCCCAGCGTTG | CTGCCACTCCGCCCTATAAG |
| SOD2 | Superoxide dismutase [Mn], mitochondrial (SOD2) | TCAGGTTGTTCACGTAGGCC | GCCCTGGAACCTCACATCAA |
| VDAC1 | Voltage-dependent anion-selective channel protein 1 (VDAC1) | CCTGCTTCTCGGCTAAAGTG | ACGTTCTTGCCATCCAGAAG |
| VDAC2 | Voltage-dependent anion-selective channel protein 2 (VDAC2) | AGATGACCTTTGACAGTCCCA | TCCTCCAAATTCTGTCCCATCA |

**SUPPLEMENTAL METHODS**

## Generation of control and patient iPSCs from fibroblasts

All study participant provided informed consent. The University of Regensburg’s ethics committee approved the study (ref: 13-101-0271). Skin biopsies were conducted by the Department of Dermatology, Regensburg University Hospital, Germany.

Fibroblasts were reprogrammed to iPSCs using the episomal protocol described in ^1^. Briefly, 5x10^5^ fibroblasts were transfected with 600 ng of each the episomal vectors pCBX-EBNS, pCE-hsk, pCE-hUL, pCE-hOCT3/4 and pCE-mp53DD using the Amaxa Nucleofactor (Lonza). The cells were then cultured in TeSR-E7 medium on Matrigel-coated dishes (Corning) until colonies appeared. iPSC colonies were manually picked and cultured on Matrigel with mTeSR1 medium.

## iPSC differentiation to NPCs and neuronal differentiation

iPSCs were differentiated to neural progenitor cells (NPCs) according to a monolayer culture protocol described in ^2^. Small iPSCs colonies were plated on Matrigel-coated plates in Neural Induction Medium (Neurobasal Medium, 2% Neural Induction Supplement, 0,5% Penicillin/Streptomycin). After 7 days, the differentiating cells were dissociated using Accutase (Life Technologies), filtered (ø50 μm) and further cultured in Neural Expansion Medium (Neurobasal/Advanced DMED F12, 2% Neural Induction Supplement, 0,5% Penicillin/Streptomycin) on Geltrex-coated plates. A pure culture of mature NPCs was obtained after 5 passages.

For neuronal differentiation, 3.5x104 NPCs from passage 5 to 12 onto polymer imaging μ-dishes (Ibidi) coated with 20% poly-L-ornithine in PBS and 43 μg/mL laminin in DMEM/F12, both overnight at 37°C. Differentiation was initiated on the next day by changing the medium to Neurobasal medium with 1% B27, 0.5% GlutaMax, 0.5% non-essential amino acids, 0.5% Culture One (Thermo Fisher Scientific), 20 ng/mL BDNF and GDNF (PeproTech), 200 nM ascorbic acid (Carl Roth 1 mM), dibutyryl-cAMP (Stemcell), 4 μg/mL laminin (Sigma) and 50 U/mL penicillin, 50 μg/mL streptomycin (Thermo Fisher Scientific). Half of the medium was changed every 3 to 4 days during the 21 days of differentiation. Proliferating cells were removed by treating the cultures with the mitotic inhibitor cytarabine (Biomol) at 1 μM from day 5 to day 6 or 7.

## Astrocytes differentiation

Astrocytes were differentiated from NPCs following a method adapted from ^3^ and used from day 30 to day 60 of differentiation 3x10^4^ NPCs were carefully dissociated and plated on Matrigel-coated plates in astrocytes media containing 2% FBS, 1% astrocytes growth supplement and 1% penicillin/streptomycin solution (ScienCell). When confluent, cells were detached and 3x10^4^ cells were seeded in new wells and this operation was repeated during the 30 days of differentiation. Immunostainings of astrocytic markers (GFAP, S100β, ALDH1L1, EAAT1, connexin 43) allowed to confirm the maturity and identity of the astrocytes. Astrocytes were grown on Matrigel-coated plates and used until day 60.

## RNA Isolation, Reverse Transcription, and Quantitative Real-Time RT-PCR

Total RNA was extracted using the RNA Plus Kit (Macherey-Nagel, Düren, Germany) according to the manufacturer’s instructions. First strand cDNA synthesis was performed from 1 μg of total RNA with QuantiTect Reverse Transcription Kit (Qiagen, Hilden, Germany). Quantitative RT-PCR experiments were performed with Rotor-Gene-Q machine (Qiagen, Hilden, Germany) using the 1x Takyon SYBR Master Mix (Eurogentec, Köln, Germany), and specific intron-spanning primers, listed in Supplemental Table S1. Measurements were performed in triplicate and results were analyzed with a Rotor-Gene-Q software version 2.3 (Qiagen, Hilden, Germany) applying the ΔΔCt method for relative quantification.

***Serotonin and Serotonin receptor 5-HT_2A_ antagonist treatments***

Serotonin hydrochloride (Sigma) was resuspended in water to a stock concentration of 100 mM and sterile filtered. M100907 (volinanserin) (Sigma-Merck), a specific 5-HT_2A_ receptor antagonist was resuspended in DMSO to a stock concentration of 10 mM.

Cells were treated with either 100 µM Serotonin alone, or with a combination of 10 µM M100907 and 100 µM Serotonin, or with 1:1000 DMSO and 100 µM Serotonin for 6 days. Medium was refreshed after 3 days.

## Analysis of mitochondrial respiration

Mitochondrial respiration was analyzed using Seahorse XFp Flux analyzer with a Seahorse XFp Mito Stress Test Kit (Agilent Technologies) according to manufacturer´s recommendations. The protocol consists in the sequential injection of 1 µM oligomycin, 2 µM FCCP, and each 0.5 µM rotenone/antimycin A (Biomol) to measure the oxygen consumption rates (OCR) of different respiratory states. The day prior the assay, 3x10^4^ astrocytes were grown in Matrigel-coated XFp 8-well miniplates. OCR measurements were normalized to the cell number.

## Immunofluorescence

Immunofluorescence stainings were performed as described in ^4^. We used the following primary antibodies: anti-β-III-tubulin (mouse; 1:2000, G7121, Promega,), anti-MAP2 (chicken; 1:5000, ab5392, Abcam), anti-VGLUT1 (rabbit; 1:500, ab180188, Abcam), anti-Neun (rabbit; 1:500, ab177487, Abcam), anti-GFAP (mouse, 1:400, C53893, Sigma-Aldrich), anti-ALDH1L1 (rabbit, 1:500, ab87117, Abcam), anti-S100β ( mouse, 1:1000, S2532, Sigma), anti-EAAT1 (rabbit, 1:200, ab416-1001, Abcam), and anti-Connexin 43 (mouse, 1:500, 14-4759-82, Invitrogen). Secondary antibodies used were: anti-mouse Cy3 (1:1000; Thermo Fisher Scientific), anti-rabbit 488, anti-chicken Cy5 (1:1000, Abcam). DAPI (1:1000; Sigma-Aldrich) stained the nuclei and coverslips were mounted with Dako Fluorescing Mounting Medium.

## Luminescent assay for ATP content

We used CellTiter-Glo®Cell Viability Kit (Promega) to measure ATP content according to manufacturer’s instructions. 1x10^5^ astrocytes were pelleted and stored at -20 °C. Cell pellets were resuspended in 500 µL PBS, heated at 100 °C for 2 min and kept on ice. Triplicates of 50 µL of sample or standard were applied to a 96-well-plate with 50 µL of CellTiter-Glo®Reagent. Luminescence was measured at an integration time of 1 sec. ATP concentrations were calculated using a 1 nM to 10 µM standard curve and normalized to protein content (µg/mL) using a BCA assay (Thermo Fisher Scientific).

## Imaging of mitochondrial membrane potential (JC-1) cytosolic Ca^2+^ (Fura-2/AM) and mitochondrial Ca^2+^ (Rhod-2/AM)

Live-cell imaging experiments were performed using Zeiss Axio Observer Z.1 microscope equipped with a Fluar 40/1.3 objective lens (Zeiss) with a 40X oil immersion objective and recorded with an AxioCam MRm CCD camera (Zeiss). Both were controlled using the ZEN 2012 imaging software. The Lambda DG-4 high-speed wavelength switcher (Sutter Instruments) was used for illumination. Analyses were performed on ImageJ (version 2.9.2). Macros were used to ensure the repeatability of the analysis. Regions of interest were drawn manually around the cells, background signal was subtracted and, where applicable, ratios were calculated. Additionally, cell size was measured in Fura-2/AM-loaded cells.

The day before experiments 1.5x10^5^ astrocytes were plated on Matrigel-coated glass coverslips. For neurons, 3.5x10^4^ NPCs were plated, and differentiated for 21 days on PLO/laminin-coated Ibidi dishes. For MMP measurement, cells were loaded with JC-1 at a concentration of 300 nM in astrocytes, and 1 μM in neurons. For calcium measurements, cells were loaded with 2 μM Fura-2/AM and 2 μM Rhod-2/AM in OptiMem. Cells were incubated with the dyes at 37 °C for 30 min.

JC-1 fluorescence was measured at 537/42 nm (green) and 620/60 nm (red), after excitation at 480/36 nm. In neurons, neurites and somas were imaged separately, as they appeared on different focus planes. Fura-2 fluorescence was measured at 510 nm after excitation at 340 or 380 nm. Rhod-2 fluorescence was measured at 576 nm after excitation at 556 nm. In neurons, after basal cytosolic Ca^2+^ measurements, spontaneous Ca^2+^ fluctuations were recorded over 20 min with 2 Hz frequency using the Fluar 20X/0.75 objective lens. Peaks were analyzed with the software IGOR Pro 9 (WaveMetrics).

## Electrophysiology

Whole-cell patch-clamp recordings were performed during the 4th week of neuronal differentiation. Micropipettes were made by pulling borosilicate glass capillaries (Science Products) using a horizontal pipette puller (Zeitz Instruments), and they were fire-polished to obtain a series resistance of 3-5 MΩ. Micropipettes were filled with intracellular solution (140 mM KCl, 1 mM MgCl2, 0.1 mM CaCl2, 5 mM EGTA, 10 mM HEPES). The extracellular solution was composed of 140 mM NaCl, 5 mM KCl, 2 mM CaCl2, 1 mM MgCl2, 10 mM HEPES, and 5 mM Glucose, pH 7.3. Recordings were performed at room temperature using a HEKA Electronic EPC-10 amplifier (HEKA Electronic). The liquid-liquid junction potential was calculated to be 4 mV (LJP calculator of the pClamp software suite, Axon Instruments) but not corrected. The series resistance was assessed but not compensated. The resting membrane potential (RMP) and capacitance were recorded directly after reaching the whole-cell configuration. For voltage-clamp recordings, membrane potential was held at -80 mV and depolarized in steps of 10 mV to evoke voltage-activated Na+- and K+-channels. Spontaneous post-synaptic currents were recorded at a holding potential of –80 mV. In current-clamp mode, manually adjusted currents were injected to hyperpolarize the membrane potential to about -80 mV and record spontaneous action potentials. Data were analyzed using Patchmaster Next (HEKA Electronic). Cells with RMP of 0 mV and above were excluded from the analysis.

## Statistical analysis

Graphical depiction and statistical analysis were conducted with Graph Pad Prism 9.5.1 (GraphPad Software). For all experiments, except patch-clamp recordings, the means three technical replicates were calculated, and three biological replicates were averaged. A technical replicate refers to the repetition of the same experimental procedure multiple times on the same sample. Biological replicates refer to independent samples. Statistical outliers were detected and eliminated using ROUT-Method.

Seahorse measurements were conducted pairwise allowing direct comparison and a mixed-effect analysis one-way ANOVA was used.

Imaging, ATP assay and patch-clamp experiments were analyzed using a one-way ANOVA without matching or pairing.

qPCR experiments data were analyzed using unpaired t-test with Welch’s correction.

Results were presented as mean ± SEM. p-value limit for statistical significance was set to ≤ 0.05.

1. Okita K, Matsumura Y, Sato Y, Okada A, Morizane A, Okamoto S *et al.* A more efficient method to generate integration-free human iPS cells. *Nat Methods* 2011; **8**(5)**:** 409-412.

2. Yan Y, Shin S, Jha BS, Liu Q, Sheng J, Li F *et al.* Efficient and rapid derivation of primitive neural stem cells and generation of brain subtype neurons from human pluripotent stem cells. *Stem Cells Transl Med* 2013; **2**(11)**:** 862-870.

3. Tcw J, Wang M, Pimenova AA, Bowles KR, Hartley BJ, Lacin E *et al.* An Efficient Platform for Astrocyte Differentiation from Human Induced Pluripotent Stem Cells. *Stem Cell Reports* 2017; **9**(2)**:** 600-614.

4. Triebelhorn J, Cardon I, Kuffner K, Bader S, Jahner T, Meindl K *et al.* Induced neural progenitor cells and iPS-neurons from major depressive disorder patients show altered bioenergetics and electrophysiological properties. *Mol Psychiatry* 2022.
